# Adaptive level modification via player skill classification and large language models

**DOI:** 10.1038/s41598-026-63084-z

**Published:** 2026-07-28

**Authors:** Ahmed A. Elshamy, Hazem N. Aliedin, Shehab T. Shaban, Moaaz H. Aldakar, Ahmed B. Zaky

**Affiliations:** 1 Department of Computer Science and Information Technology, Egypt-Japan University of Science and Technology (E-JUST), New Borg El Arab City, Alexandria Egypt; 2 Department of Electrical Engineering, Faculty of Engineering (Shoubra), Benha University, Banha, Qalubiya Egypt

**Keywords:** Game generation, Game development, Procedural content generation, Player behavior, Large language models, Engineering, Mathematics and computing

## Abstract

Maintaining player engagement in video games requires a careful balance between challenge and player competence. Static difficulty settings fail to account for individual skill variation, while existing dynamic difficulty adjustment systems are limited to tuning low-level game parameters rather than restructuring level content. This paper presents an adaptive level modification framework that personalizes gameplay by continuously inferring player skill and applying targeted structural modifications to level content in real-time. A hybrid behavioral dataset is constructed by combining agent-generated trajectories, produced by Proximal Policy Optimization (PPO) agents, a reinforcement learning approach, trained at three distinct skill levels, with manually collected human gameplay data labeled through clustering. A classifier trained on this dataset categorizes players into expert, normal, and beginner skill levels, achieving an overall accuracy of 97.82%. The classifier output drives a two-stage large language model (LLM) pipeline guided by prompt engineering, which expands a skill-conditioned prompt into a structured modification instruction applied to the current level chunk. A physics-constrained verifier based on a graph-based shortest path method ensures all modified levels remain traversable. Evaluated on Super Mario Bros. levels, the framework achieves a post-modification playability rate of 74.1% at the full-level granularity and 83.5% at the isolated-chunk granularity, closely matching the 80.0% baseline of the original levels.

## Introduction

Video games are designed to be engaging, but engagement is inherently personal. A level that feels perfectly challenging to one player may feel trivially easy to another, or overwhelmingly difficult to a third. This mismatch between a player’s skill level and the difficulty of the game content is one of the most persistent sources of disengagement in interactive entertainment. When a game is too easy, it becomes monotonous; when it is too hard, it becomes discouraging. In either case, the player loses the sense of immersion and engagement that defines genuinely enjoyable gameplay – a state that requires a careful and continuous balance between challenge and competence. This balance is not static; it shifts as the player learns, adapts, and improves over the course of a session. A game that succeeds in maintaining this balance over time must therefore be capable of recognizing and responding to changes in player behavior, rather than relying on a fixed, one-size-fits-all configuration of challenge.

Traditional game design addresses this through static difficulty settings, where the player selects a predefined difficulty tier before play begins. While simple, this approach is coarse and inflexible, requiring the player to self-assess their own skill and offering no mechanism for adapting to changes in performance over the course of a session. A player who selects a difficulty tier that turns out to be mismatched with their actual skill level has no recourse other than restarting the game under a different setting, breaking immersion and interrupting the play experience. Dynamic difficulty adjustment (DDA) was proposed as a principled response to this limitation^1^, enabling game systems to monitor player behavior and adjust challenge in real time. However, most existing DDA systems operate by tuning low-level game parameters such as enemy health, spawn rates, or item availability, rather than modifying the structural content of the level itself. This limits their expressiveness and leaves the spatial and navigational experience of the game largely unchanged regardless of the player’s skill. A player who struggles with platforming challenges, for instance, will continue to face the same geometry and layout regardless of how many in-game parameters are adjusted around them.

Procedural content generation (PCG) offers a complementary perspective, providing tools for automatically constructing or modifying game levels^2^. By delegating the design of level content to an automated process, PCG enables a degree of variability and adaptability that is difficult to achieve through manual design alone. Recent advances in large language models (LLMs) have opened new possibilities in this space, with systems such as MarioGPT^3^ demonstrating that pretrained language models can generate coherent and playable platformer levels from natural language prompts. These developments suggest that LLMs are capable of understanding and manipulating the structural conventions of game levels in a semantically meaningful way. However, these approaches focus on content generation in isolation, without grounding the generation process in a model of the player or connecting it to an adaptive feedback loop that responds to observed gameplay behavior. The result is a system that can produce varied content but cannot tailor that content to the individual playing it.

The remainder of this paper is organized as follows. “Related work” section reviews related work on LLM-based procedural content generation, classical PCG methods, and data-driven player modeling and dynamic difficulty adjustment. “Proposed approach” section describes the methodology in detail, covering the construction of the hybrid behavioral dataset combining agent-generated and human gameplay trajectories, the data representation and preprocessing pipeline, the classification step, the prompt-based adaptive level modification pipeline, the two-stage LLM generative approach guided by prompt engineering, and the physics-constrained pathfinding verification procedure. “Results” section presents the experimental results, including the clustering analysis, the classification performance, the playability verification outcomes across both the original and modified levels, and a structural design metric analysis demonstrating statistically significant alignment between modified chunk properties and targeted skill labels. “Discussion” section discusses the findings and their implications, and acknowledges the limitations of the current approach. “Conclusion” section concludes the paper with a summary of the key contributions and findings.

## Related work

Recent work has investigated the use of large language models (LLMs) for procedural game content generation by representing game artifacts as token sequences. MarioGPT formulates 2D platformer level generation as an autoregressive sequence modeling problem, enabling a pretrained GPT-style model to generate playable Super Mario Bros. levels. The method incorporates natural language prompt conditioning via cross-attention, allowing intuitive control over level characteristics, and integrates novelty search to support open-ended and diverse level generation^3^. Game Generation via Large Language Models extends this paradigm by using a single pretrained LLM to generate both game rules and levels. By employing a structured video game description language and prompt-based constraints, the model produces coherent rule sets alongside compatible level layouts, highlighting the potential of LLMs for modeling hierarchical and relational game structures in addition to spatial content^4^. Beyond game content, LLMs have also demonstrated strong performance in structured classification tasks, with recent work showing that they can effectively categorize complex, high-dimensional inputs into discrete classes^5^. However, these approaches treat content generation as a standalone process, operating independently of any model of the player and without a mechanism for conditioning generation on observed gameplay behavior. As a result, the generated content is not grounded in the needs or abilities of the individual player, limiting its applicability in adaptive gameplay contexts.

Prior to the rise of LLM-based approaches, procedural content generation (PCG) for platform games was predominantly addressed through search-based and machine learning methods. The taxonomy and foundations of search-based PCG were established by framing level generation as an optimization problem in which candidate content is evaluated and refined through evolutionary or heuristic search^2^. A subsequent survey of PCG via machine learning catalogued approaches ranging from Markov models to deep generative networks, establishing the empirical and theoretical benchmarks against which learned generators are measured^6^. While these methods offer principled and well-studied solutions for content generation, they are predominantly designed for offline generation pipelines and require either carefully engineered fitness functions or large corpora of training levels. This makes real-time, player-conditioned content modification difficult to achieve within these paradigms. The approach presented in this work departs from these methods by delegating content transformation to a language model guided by structured natural language prompts, bypassing the need for an explicit fitness function or a learned generative model trained on level corpora.

Player modeling is a central component of any adaptive game system. A comprehensive treatment of data-driven player modeling covers behavioral feature extraction, experience-driven content generation, and the use of supervised and unsupervised methods to infer player state from gameplay traces^7^. More directly related to the classification task in this work, a systematic review of data-driven approaches to player modeling identifies clustering and multi-class classification as the dominant paradigms for inferring player skill and behavior type from in-game logs^8^. Behavioral recognition from interaction data has also been explored in non-game contexts, with activity recognition systems demonstrating that structured behavioral features extracted from sensor and visual data can reliably distinguish between different performance profiles^9^. Despite the maturity of these techniques, their integration into end-to-end adaptive content pipelines remains limited, with most systems treating player modeling and content generation as separate and loosely coupled components. The work presented here contributes to this line of research by constructing a hybrid behavioral dataset that combines agent-generated and human gameplay trajectories, and training a classifier on behavioral state features to categorize players into discrete skill levels, with the classifier output directly driving the content modification stage.

The broader motivation for the proposed approach lies in the field of dynamic difficulty adjustment (DDA), which seeks to maintain player engagement by aligning game challenge with player skill in real time. The foundational argument for DDA demonstrates that adaptive systems can improve player engagement without disrupting immersion, with an early inventory-based implementation presented in a first-person game environment^1^. A comprehensive review of DDA techniques in computer games covers rule-based, reinforcement learning, and neural approaches, identifying the lack of robust player skill inference as a persistent bottleneck^10^. Across these approaches, difficulty modulation is predominantly achieved by adjusting numerical game parameters such as enemy speed, damage values, or resource availability, rather than altering the spatial structure of the level itself. This means that the navigational and platforming experience of the game remains structurally unchanged regardless of the player’s skill, leaving a significant dimension of the gameplay experience unaddressed. The work presented here addresses this gap by incorporating an explicit skill classifier as the core decision module, and delegates difficulty modulation to a prompt-based LLM pipeline that modifies level structure rather than adjusting game parameters.

## Proposed approach

**Fig. 1:**
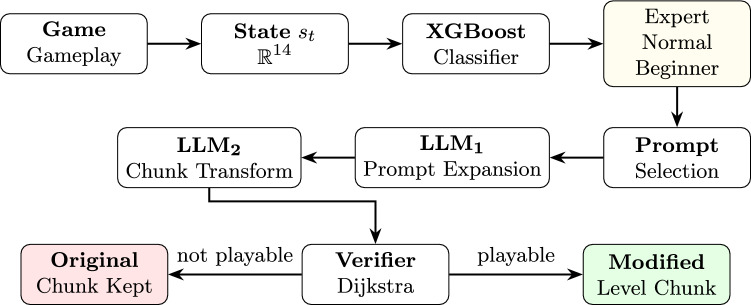
Overview of the proposed adaptive level modification pipeline.

This section presents the proposed adaptive level modification pipeline, which integrates player behavior classification with prompt-based LLM level modification. The approach continuously monitors gameplay through a structured state representation and classifies the player into one of three skill levels – expert, normal, or beginner – using a classifier trained on a hybrid dataset of agent-generated and human gameplay trajectories. Based on the inferred skill level, structural modifications are applied to the current level chunk through a two-stage LLM pipeline guided by prompt engineering. A physics-constrained verifier based on Dijkstra’s algorithm then ensures all modified levels remain traversable before being accepted. Together, these components form a system that adapts the spatial structure of the game environment to the player in real time, delivering a genuinely personalized gameplay experience (see Fig. 1).

The diagram depicts the sequential flow of information through the system: the game environment produces a structured state representation at each timestep, which is passed to the classifier to infer the player’s skill level. The predicted label is then used to select an appropriate modification prompt, which is expanded and applied to the current level chunk through the two-stage LLM pipeline. The resulting modified chunk is subsequently passed to the verifier, which determines whether it remains traversable. If the modified chunk is deemed playable, it replaces the original; otherwise, the original chunk is retained unchanged.

### Data collection and description

#### The Mario dataset

The level data used in this work is sourced from the Video Game Level Corpus (VGLC)^11^, a publicly available collection of human-designed levels from classic video games. Specifically, we use the complete Super Mario Bros. subset, which comprises all 15 available levels. Each level is represented as a 2D grid of discrete tiles, where each tile encodes a distinct game element such as air (-), solid ground (X), coins (o), or enemies and hazards (E). The levels are stored as .txt files, with tile annotations remaining consistent across all levels, allowing the model pipeline to interpret and modify level content in a reliable and structured manner. The dataset provides a diverse range of level layouts and obstacle configurations, making it a representative and reliable basis for experimentation.

#### The player behavior dataset

This subsection describes the procedure used to construct the player behavior dataset. The environment is implemented as a Super Mario-like game in Godot Engine, with which the reinforcement learning agents interact to collect gameplay trajectories. To capture a realistic and diverse range of gameplay styles, agent trajectories are generated using Proximal Policy Optimization (PPO)^12^, where each agent is trained to a specific skill level determined by its total training duration. The resulting trajectories are recorded alongside manually collected human gameplay sessions, labelled according to their corresponding skill level, and aggregated into a single consolidated dataset suitable for supervised classification of player skill.

***Proximal policy optimization (PPO)***

Player behavior trajectories are sequential records of gameplay interactions, capturing the state observations, actions, and rewards experienced by an agent over the course of a play session. These trajectories serve as the primary source of behavioral data from which player skill is inferred, encoding how a player navigates the environment, responds to obstacles, and progresses through the level over time. In this work, trajectories are generated using Proximal Policy Optimization (PPO)^12^ as the underlying reinforcement learning algorithm. PPO is a policy-gradient method that updates a stochastic policy $\pi _\theta (a|s)$ while limiting the change from the current policy $\pi _{\theta _\text {old}}$ to ensure stable learning, making it well suited for complex and dynamic environments such as platformer games where decision making requires both precise control and long-term planning. In this work, PPO is implemented using the Stable-Baselines3 library^13^ with an MLP policy and a learning rate of $\alpha = 3 \times 10^{-4}$. The core of PPO is the clipped surrogate objective:

1 $$\begin{aligned} L^{\text {CLIP}}(\theta )&= \mathbb {E}_t \Big [ \min \big ( r_t(\theta ) A_t,\, \text {clip}(r_t(\theta ), 1-\epsilon , 1+\epsilon ) A_t \big ) \Big ] \end{aligned}$$

2 $$\begin{aligned} r_t(\theta )&= \frac{\pi _\theta (a_t \mid s_t)}{\pi _{\theta _{\text {old}}}(a_t \mid s_t)} \end{aligned}$$

where $r_t(\theta )$ is the probability ratio between the new and old policies, and $A_t$ is the advantage estimate, which measures how much better an action is compared to the expected value. In practice, $A_t$ is computed using Generalized Advantage Estimation (GAE)^14^:

3 $$\begin{aligned} A_t = \sum _{l=0}^{T-t-1} (\gamma \lambda )^l \delta _{t+l}, \quad \delta _t = r_t + \gamma V(s_{t+1}) - V(s_t) \end{aligned}$$

where *V*(*s*) is the value function, *γ* the discount factor, *λ* a smoothing parameter, and $r_t$ the reward received at time step *t*. The final PPO loss combines the clipped objective with a value-function loss $L^{\text {VF}}$ and an entropy bonus $S[\pi _\theta ]$:

4 $$\begin{aligned} L(\theta ) = L^{\text {CLIP}}(\theta ) - c_1 L^{\text {VF}}(\theta ) + c_2 S[\pi _\theta ](s_t) \end{aligned}$$

where $L^{\text {VF}}$ encourages accurate state-value predictions and $S[\pi _\theta ]$ promotes exploration by penalising overly deterministic policies.

***Skill-level definition via training duration***

Distinct player skill levels are modelled by varying the amount of training experience provided to the RL agent. Each skill level is additionally associated with a dedicated reward function that shapes the agent’s behavior to reflect the corresponding level of competence. Specifically, three behavior categories are defined based on the total number of training timesteps:

- *Expert* Trained for 300,000 timesteps with a reward function that strongly incentivises forward progress and coin collection while applying heavy penalties for death, enabling the agent to learn near-optimal navigation strategies, precise jump timing, and consistent obstacle avoidance.
- *Normal* Trained for 100,000 timesteps with a reward function that balances progress with survival incentives and mild exploration bonuses, resulting in partially learned behavior with occasional execution errors and suboptimal decision making.
- *Beginner* Trained for 20,000 timesteps with a reward function that provides only minimal progress incentives and a small survival bonus, producing exploratory, unstable, and largely suboptimal actions with limited environmental awareness.

This controlled variation in both training duration and reward shaping induces a systematic and measurable degradation in policy quality across the three categories, allowing the collection of behaviorally diverse trajectories that span the full spectrum of player competence.

***Trajectory collection and state representation***

During interaction with the environment, the agent operates in discrete time steps. To balance data granularity and storage efficiency, transitions are recorded at a fixed interval of every 5 seconds of gameplay. At each recorded step *t*, a transition tuple is stored as:

5 $$\begin{aligned} \mathcal {D}_t = (s_t, a_t, r_t, l) \end{aligned}$$

where $s_t$ denotes the observed state, $a_t$ the executed action drawn from a discrete action space of six possible movement commands:

6 $$\begin{aligned} \mathcal {A} = \{\text {Idle},\, \text {Left},\, \text {Right},\, \text {Jump},\, \text {Crouch},\, \text {Run}\}, \end{aligned}$$

$r_t$ the received reward, and $l \in \{\text {expert}, \text {normal}, \text {beginner}\}$ the corresponding skill-level label. All transitions are aggregated into a single consolidated dataset.

The state vector $s_t$ captures kinematic information, environmental context, and temporal progression, and is defined as:

7 $$\begin{aligned} s_t = [v_x,\, v_y,\, d,\, f,\, c_1,\, c_2,\, j,\, k,\, g,\, e,\, i,\, \sigma ,\, t,\, x] \;\in \; \mathbb {R}^{14}, \end{aligned}$$

where each component is described in Table 1.

**Table 1:** State vector components used for trajectory collection.

Symbol	Feature name	Description
$v_x$, $v_y$	vel_x, vel_y	Horizontal and vertical velocities of the player
*d*	dir_input	Directional input
*f*	on_floor	Binary indicator of ground contact
$c_1$, $c_2$	dist_to_chunk_1/2	Distances to the next two level chunks ahead
*j*	jump_active	Binary indicator of jump action activation
*k*	crouch_active	Binary indicator of crouch action activation
*g*	is_near_gap	Proximity to a gap
*e*	enemy_in_front	Presence of an enemy ahead
*i*	chunk_index	Index of the current level chunk
*σ*	sw_rate	Action switching rate
*t*	map_time	Elapsed level time
*x*	pos_x	Horizontal position within the level

***Action, reward, and label encoding***

The control decision executed by the agent at each timestep is stored as a discrete action variable $a_t \in \mathcal {A}$, representing one of six available movement commands in the environment. Each action is evaluated using a scalar reward $r_t$ whose structure varies according to the skill level being simulated. Specifically, three reward functions are defined as follows:

- *Expert* $r_t$ strongly weights forward progress and coin collection, with a heavy penalty for death:
  $$r_t = 0.1 \cdot \text {progress} + 1.0 \cdot \text {coin} - 1.0 \cdot \text {death} - 0.05 \cdot \text {inefficiency}$$
- *Normal* $r_t$ balances progress with survival and mild exploration incentives:
  $$r_t = 0.05 \cdot \text {progress} + 0.01 \cdot \text {survival} - 0.5 \cdot \text {death} + 0.01 \cdot \text {exploration}$$
- *Beginner* $r_t$ provides only minimal progress and survival incentives with a reduced death penalty:
  $$r_t = 0.001 \cdot \text {progress} + 0.005 \cdot \text {survival} + 0.002 \cdot \text {switching} - 0.2 \cdot \text {death}$$

Each transition is additionally annotated with a categorical skill-level label:

8 $$\begin{aligned} l \in \{\text {expert}, \text {normal}, \text {beginner}\} \end{aligned}$$

where the label is assigned based on the training duration of the agent that produced the trajectory, as defined in the previous paragraph.

To further enrich the dataset, additional trajectories were collected through manual human gameplay sessions, where players deliberately mimicked each skill level using the same state representation and action space as the agent-generated data. Since these sessions carry no automatic skill-level labels, a clustering step was applied to assign them, as described in the following “Data representation and preprocessing” section. The labeled human trajectories were then merged with the agent-generated trajectories to form the final consolidated dataset, capturing a broad spectrum of gameplay behaviors ranging from exploratory and suboptimal to near-optimal and precise.

### Data representation and preprocessing

Each level in the mario dataset is segmented into six spatial chunks, capturing the structural organization of the game environment. These chunks are represented as nodes in a linked list, preserving their sequential order within the level. Each node contains a symbolic representation of the chunk, describing the arrangement of tiles, obstacles, and interactive elements.

**Fig. 2:**
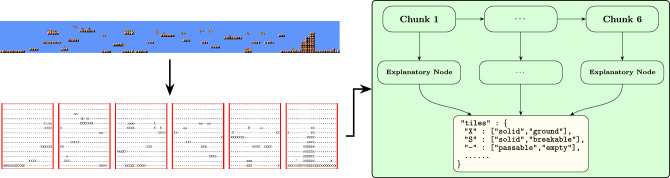
Image segmentation into semantic chunks, each annotated with interpretable labels defined in a shared schema.

To encode additional semantic information, each chunk node is accompanied by a supplementary explanatory node that defines the properties of the symbols within the chunk, including their functional meaning and associated probabilities. Together, these nodes form a graph-like structure stored in JSON format to facilitate programmatic access and interoperability. In this representation, each level is encoded as a top-level object containing sequential chunk nodes, where each node includes a nested object representing its explanatory information, as illustrated in Fig. 2.

During preprocessing, the JSON structure is parsed to convert symbolic tile representations into a machine-readable format suitable for model input. Additional preprocessing steps include normalizing features, verifying consistency across chunk and explanatory nodes, and ensuring that all semantic properties are correctly represented. This structured representation preserves both the spatial relationships between level segments and the semantic meaning of individual tiles, enabling effective use in the generative modeling pipeline.

#### The player behavior dataset

***Dataset construction and preprocessing***

Player behavior is modeled as a discrete-time state sequence, where each recorded time step is represented by the state vector $s_t$ defined in “The player behavior dataset” section. This state-based formulation provides a compact and consistent abstraction of player behavior over time, and serves as the primary input representation used by the classification component of the proposed framework. Prior to training, several preprocessing steps are applied to ensure data quality and learning stability. The agent-generated trajectories and the manually collected human gameplay trajectories are loaded from their respective CSV files and concatenated into a single unified dataset. Any samples containing missing or invalid values are removed using a dropping strategy applied across all feature columns. The categorical behavior labels $l \in \{\text {expert}, \text {normal}, \text {beginner}\}$ are then encoded into numerical form as $\{0, 1, 2\}$ respectively, to support multi-class classification. Finally, the dataset is partitioned into training and testing subsets using stratified sampling, which preserves the class distribution across both splits and ensures a fair and unbiased evaluation of the classifier.

***Labeling Human Gameplay Trajectories via Clustering***

 Unlike the agent-generated trajectories, which carry behavior labels assigned during simulation, the manually collected human gameplay trajectories contain no ground-truth skill annotations. To address this, unsupervised clustering is applied to derive behavior labels from the structure of the data itself. Specifically, K-means clustering with *k = 3* is applied to the human trajectory samples, where the number of clusters directly corresponds to the three target skill levels. Prior to clustering, all feature columns are standardized via z-score normalization:

9 $$\begin{aligned} \hat{s}^{(j)} = \frac{s^{(j)} - \bar{s}^{(j)}}{\sigma ^{(j)}} \end{aligned}$$

where $\bar{s}^{(j)}$ and $\sigma ^{(j)}$ are the mean and standard deviation of feature *j* across all samples, ensuring that no single feature dominates the distance computations due to scale differences. The algorithm then partitions the samples into clusters $\{C_1, C_2, C_3\}$ by minimizing the within-cluster sum of squared distances:

10 $$\begin{aligned} \underset{\{C_i\}}{\arg \min } \sum _{i=1}^{3} \sum _{s \in C_i} \Vert s - \mu _i \Vert ^2 \end{aligned}$$

where $\mu _i$ denotes the centroid of cluster $C_i$. Each resulting cluster is then mapped to a skill label based on the mean horizontal velocity $\overline{v}_x(\mu _i)$ of its centroid:

11 $$\begin{aligned} \text {label}(C_i) = {\left\{ \begin{array}{ll} \textit{beginner} & \text {if } \overline{v}_x(\mu _i) = \min _j\, \overline{v}_x(\mu _j) \\ \textit{expert} & \text {if } \overline{v}_x(\mu _i) = \max _j\, \overline{v}_x(\mu _j) \\ \textit{normal} & \text {otherwise} \end{array}\right. } \end{aligned}$$

This mapping reflects the observable behavioral tendency that more skilled players navigate levels at a faster and more decisive pace. Once labeled, the human trajectories are concatenated with the agent-generated dataset to form the final hybrid dataset used for classifier training.

### Classification step

The classification framework employs a gradient boosting decision tree model based on XGBoost^15^, selected for its efficiency and strong performance on structured behavioral data. Prior to training, the class distribution is balanced using the Synthetic Minority Oversampling Technique (SMOTE)^16^, which generates synthetic samples for underrepresented classes to prevent the model from being biased toward the majority class. Given an input feature vector $\textbf{x}_i \in \mathbb {R}^{12}$, comprising the kinematic and environmental features $[v_x, v_y, d, f, c_1, c_2, j, k, g, e, i, \sigma ]$ as described in Table 1, the model predicts the class probabilities using an additive ensemble of *M = 150* decision trees:

12 $$\begin{aligned} \hat{\textbf{p}}_i = \textrm{softmax} \left( \sum _{m=1}^{M} f_m(\textbf{x}_i) \right) \end{aligned}$$

where $f_m(\cdot )$ denotes the *m*-th regression tree and $\hat{\textbf{p}}_i \in \mathbb {R}^3$ represents the predicted probability distribution over the three behavioral classes (expert, normal, beginner).

Model training minimizes an objective function approximating the regularized multi-class cross-entropy loss. While the theoretical formulation includes a complexity penalty:

13 $$\begin{aligned} \mathcal {L} = - \sum _{i=1}^{N} \sum _{c=1}^{3} y_{i,c} \log \hat{p}_{i,c} + \sum _{m=1}^{M} \Omega (f_m) \end{aligned}$$

with regularization term:

14 $$\begin{aligned} \Omega (f) = \gamma T + \frac{1}{2} \lambda \Vert \textbf{w} \Vert ^2 \end{aligned}$$

where *T* is the number of leaves in the tree and $\textbf{w}$ are the leaf weights, the practical implementation relies primarily on structural constraints and stochastic regularization. Specifically, the model uses a maximum tree depth of 4 to limit model complexity, instance subsampling (subsample *= 0.8*) and feature subsampling (colsample_bytree *= 0.8*) at each boosting iteration to control overfitting, with *γ = 0* and *λ = 0* (i.e., no explicit leaf-weight or split-complexity penalties beyond defaults). A learning rate of *η = 0.05* ensures stable and gradual convergence across boosting iterations.

The trained model outputs calibrated class probabilities via the multi:softprob objective and serves as the core decision module in the behavioral adaptation pipeline.

### Prompt-based adaptive level modification

Following the classification of the player’s skill level, the framework applies targeted structural modifications to the current level chunk through a prompt-based generation pipeline. Each skill level is associated with a set of predefined natural language prompts, from which one is randomly selected at each chunk to guide the language model. For example, a chunk classified as *Expert* may receive a prompt such as *“add more enemies”* or *“introduce gaps that require precise jumping”*, while a *Beginner* chunk may receive prompts encouraging wider platforms and fewer hazards.

**Fig. 3:**
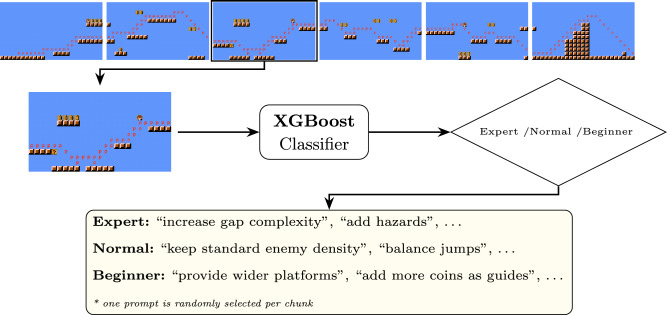
Adaptive level modification pipeline: a chunk is extracted from the level, classified by XGBoost into a skill level, and a prompt is randomly selected from the corresponding fixed prompt set to guide the level modification.

Formally, let $C_k$ denote the *k*-th chunk of the level and $l_k \in \{\text {expert}, \text {normal}, \text {beginner}\}$ the predicted skill label for that chunk. Let $\mathcal {S}_{l_k}$ denote the fixed prompt set associated with skill level $l_k$. The prompt selection function $\mathcal {P}$ randomly samples a prompt from the corresponding set:

15 $$\begin{aligned} p_k = \mathcal {P}(C_k, l_k) \sim \mathcal {U}(\mathcal {S}_{l_k}) \end{aligned}$$

where $\mathcal {U}(\mathcal {S}_{l_k})$ denotes a uniform random draw from the fixed prompt set $\mathcal {S}_{l_k}$, and $p_k$ is the selected prompt passed to the language model to produce the modified chunk $\hat{C}_k$. This process is applied independently to each chunk, allowing the framework to adaptively tailor different segments of the level to the player’s skill profile, as illustrated in Fig. 3.

### LLM-based generative framework

The proposed generative framework consists of two sequential Large Language Models (LLMs), each assigned a distinct role to ensure controlled and level-aware content transformation.

#### Prompt expansion via the first LLM

The selected prompt $p_k$ is a short, high-level directive that specifies the desired modification for the current chunk. While concise, such prompts lack the structural detail and explicit constraints needed to reliably guide a generative model toward consistent and level-appropriate outputs. To address this, the first LLM is tasked with prompt refinement and expansion.

Specifically, the first LLM receives three inputs: the original symbolic chunk $C_k$, the selected prompt $p_k$, and a set of predefined system-level writing guidelines $\mathcal {G}$ that define the expected output format, stylistic requirements, and transformation constraints. The model then expands $p_k$ into a comprehensive and structured prompt $\hat{p}_k$:

16 $$\begin{aligned} \hat{p}_k = \text {LLM}_1(C_k,\, p_k,\, \mathcal {G}) \end{aligned}$$

In this work, $\text {LLM}_1$ is instantiated as Devstral Medium^17^, a code-capable instruction-following model developed by Mistral AI, accessed via the Mistral API with a temperature of 0.3 to ensure deterministic and reproducible outputs. The model is well suited for structured prompt expansion owing to its strong instruction-following capability and precise text manipulation behaviour.

The expanded prompt $\hat{p}_k$ incorporates explicit modification objectives, structural constraints aligned with the predicted skill level $l_k$, and clearly defined output expectations. This expansion step standardizes and formalizes the editing instructions before they are applied to the symbolic chunk, improving consistency, controllability, and reproducibility across different chunks and skill levels. The system-level guidelines $\mathcal {G}$ used in this stage are detailed in Table 2.

**Table 2:** System-level guidelines used by the first LLM for prompt expansion.

#	Guideline
1	Specify exact changes to make, including which symbols to replace and with what.
2	Reference specific row numbers to modify.
3	State explicitly how many enemies or obstacles to add or remove.
4	Pipes ($\texttt {< >}$ top, [ ] body) must be treated as a single block. If moved or removed, all pipe tiles must be moved or removed together, and vacated tiles replaced with-.
5	All changes must occur along the player’s path. Coins (o) may only be placed within the player’s reachable range. Enemies (E) must always stand on solid ground (X) and cannot be placed in the air. The level must remain fully playable.
6	Ground tiles (X) may only be removed by replacing them with air (-). Ground tiles cannot be replaced with any other tile type. Gaps created with- must not break level playability.
7	Question blocks (? or Q) must always be placed in the air above the player’s path so the player can hit them from below by jumping. They cannot be placed on the ground or inside solid structures.
8	Cannons consist of B (top) and b (bottom) and must be treated as a single 2-tile block. The bottom (b) must rest on the player’s path with the top (B) directly above it. Neither tile may be moved separately, and cannons must not float or overlap other structures.
9	Apply the following design philosophy according to the target skill level: **Beginner** prioritises safety by using continuous ground, removing all hazards, and placing coins as navigational guides; **Normal** applies standard mechanics by using isolated hazards and simple jumps that are easy to time; **Expert** enforces precision and pressure by clustering hazards together and using gaps or narrow pillars to restrict landing options.

An example of the prompt expansion process is illustrated in Fig. 4, showing how a short skill-conditioned directive is transformed into a structured and detailed modification instruction.

**Fig.Fig. 4:**
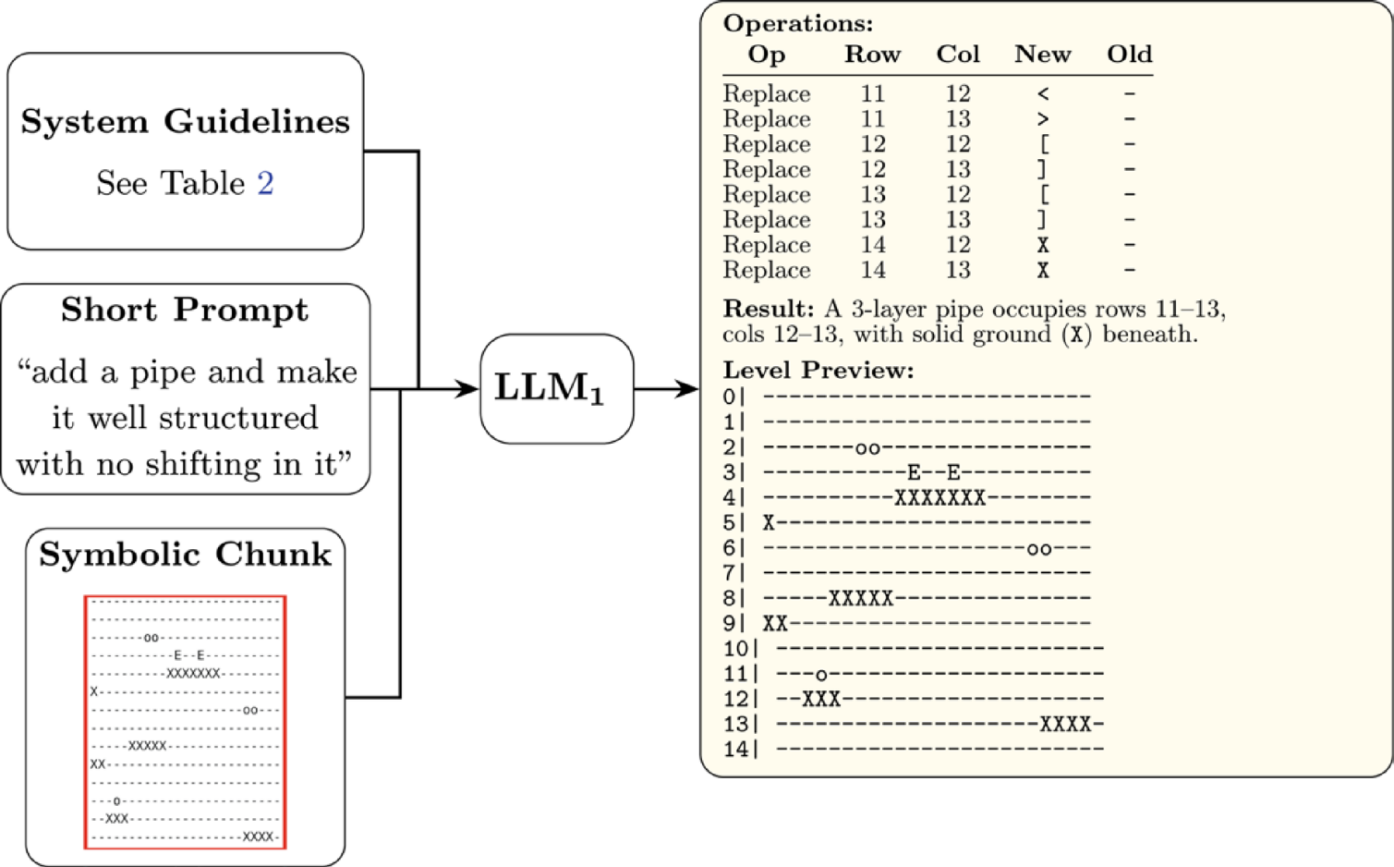
Prompt expansion pipeline: the first LLM receives system guidelines, a short skill-conditioned prompt, and the symbolic chunk, and produces a structured set of editing operations with a level preview.

#### Chunk transformation via the second LLM

**Fig. 5:**
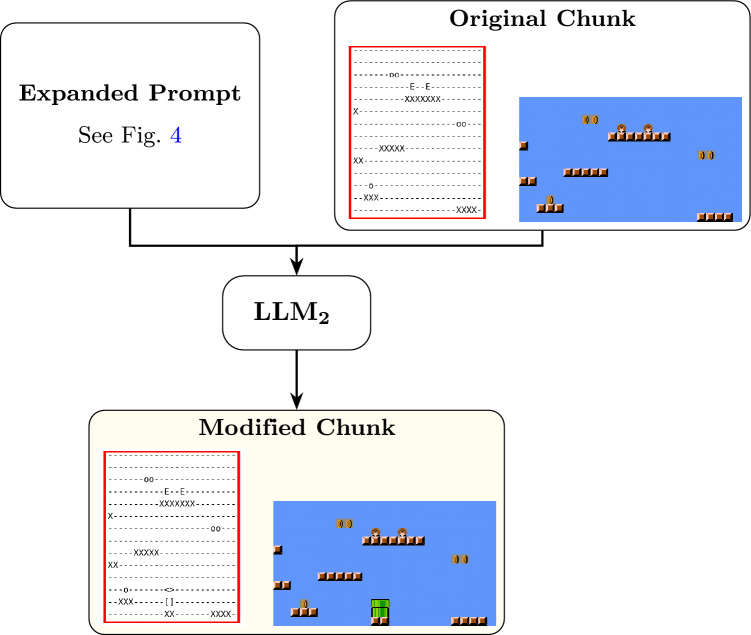
The second LLM receives the expanded prompt and the original symbolic chunk, and produces the final modified chunk.

In the final stage, the original symbolic chunk $C_k$ and the expanded prompt $\hat{p}_k$ produced by $\text {LLM}_1$ are passed together to a second language model $\text {LLM}_2$, which performs the actual content transformation:

17 $$\begin{aligned} \hat{C}_k = \text {LLM}_2(C_k,\, \hat{p}_k) \end{aligned}$$

where $\hat{C}_k$ denotes the modified chunk. The model applies the structured editing instructions in $\hat{p}_k$ to transform the symbolic tile layout of $C_k$, producing a modified chunk that reflects the target skill level while preserving the core structure and semantic content of the original. The same model architecture is used for both stages, with $\text {LLM}_2$ instantiated as Devstral Medium^17^, ensuring consistency across the pipeline. An example of the resulting modified chunk is shown in Fig. 5.

This two-stage architecture deliberately separates prompt engineering from content transformation. The first stage standardizes and formalizes the modification intent, while the second stage focuses exclusively on applying those instructions to the symbolic chunk. This separation improves modularity, interpretability, and control over the generation process, and reduces the risk of ambiguous or inconsistent edits that may arise from passing short, underspecified prompts directly to a generative model. Once the modified chunk $\hat{C}_k$ is produced, it replaces the original chunk $C_k$ in the level map, reconstructing the full level with the targeted modification applied while leaving all other chunks unchanged.

### Playability verification via physics-constrained pathfinding

To ensure that any generated level chunk, once integrated into the full map, remains traversable by the player, a formal playability verification procedure is introduced. The procedure models physically plausible player movement as a directed weighted graph, where nodes represent passable tiles and edges encode valid transitions under simplified platformer physics. Reachability analysis is then performed using Dijkstra’s algorithm^18^ to determine whether a path exists from any valid starting position to the level’s goal. This provides a principled and deterministic mechanism for filtering non-traversable level configurations before they are accepted as valid output.

#### Physics-constrained movement model

The symbolic level map is represented as a discrete 2D grid $\mathcal {M} \in \Sigma ^{H \times W}$, where *H* and *W* denote the height and width of the map in tiles, and *Σ* is the tile alphabet encoding terrain features such as solid blocks, air, pipes, and enemies. Each tile $M_{i,j} \in \Sigma$ at row *i* and column *j* is classified as either *solid* or *passable* according to:

$$\phi (M_{i,j}) = {\left\{ \begin{array}{ll} 1 & \text {if } M_{i,j} \in \Sigma _{\text {solid}}, \\ 0 & \text {otherwise}, \end{array}\right. }$$

where $\Sigma _{\text {solid}} \subset \Sigma$ is the set of solid tile types. A tile is considered *passable* if $\phi (M_{i,j}) = 0$, and a player occupying tile (*i*, *j*) is said to be *grounded* if it is passable and the tile directly beneath it is solid:

$$\text {grounded}(i, j) \;\triangleq \; \phi (M_{i,j}) = 0 \;\wedge \; \phi (M_{i+1,j}) = 1.$$

To model feasible player transitions, we define a deterministic transition function $\mathcal {T}$ that encodes three classes of movement primitives under simplified platformer physics:

- *Walking* A grounded player at (*i*, *j*) may move horizontally to *(i, j ± 1)* if the target tile is passable and no solid tile lies along the horizontal path between them.
- *Falling* A player at (*i*, *j*) with no solid tile beneath it transitions downward to $(i+1,\, j + \delta _c)$ for $\delta _c \in \{-1, 0, 1\}$, subject to passability of the target tile and the absence of any solid tile along the short diagonal trajectory, where $\delta _c$ denotes the horizontal drift during the fall, allowing the player to land directly below or on either adjacent diagonal tile.
- *Jumping* A grounded player at (*i*, *j*) may jump to a target tile $(i + \Delta r,\, j + \Delta c)$, where $\Delta r \in [-4, -1]$ and $\Delta c \in [-4, 4] \setminus \{0\}$, subject to the following conditions: (i) the target tile is passable, (ii) the tile directly above the player is not solid, and (iii) every tile along the linear trajectory from source to target is passable. The jump cost is defined as:
  $$\omega _{\text {jump}}(\Delta r, \Delta c) = 3 + |\Delta r| + |\Delta c|,$$
  reflecting the physical effort of longer or higher jumps.

Condition (iii) is enforced by linearly interpolating between the source tile (*i*, *j*) and the target tile $(i + \Delta r,\, j + \Delta c)$ at integer steps, verifying that no intermediate tile is solid. Formally, for a trajectory of $s = \max (|\Delta r|, |\Delta c|)$ steps, each intermediate tile at step $t \in \{1, \dots , s\}$ is given by:

$$\left( \Bigg \lfloor i + \frac{\Delta r \cdot t}{s} \Bigg \rceil ,\; \Bigg \lfloor j + \frac{\Delta c \cdot t}{s} \Bigg \rceil \right) ,$$

where $\lfloor \cdot \rceil$ denotes rounding to the nearest integer. A move is considered blocked if any tile along this sequence is solid, ensuring that no trajectory passes through walls or ceilings. In practice, this traversal is implemented using Bresenham’s line algorithm^19^, which guarantees that diagonal corners shared between two tiles are never skipped, preventing any trajectory from passing through a one-tile-wide wall.

Formally, the transition function $\mathcal {T}$ maps a source tile and a displacement to a feasibility indicator and an associated traversal cost:

$$\mathcal {T}\bigl ((i,j),\, (\Delta r, \Delta c)\bigr ) \;=\; {\left\{ \begin{array}{ll} \bigl (1,\; \omega \bigr ) & \text {if the move is physically feasible,} \\ \bigl (0,\; \infty \bigr ) & \text {otherwise,} \end{array}\right. }$$

where $\omega \in \mathbb {R}_{>0}$ is the traversal cost associated with the movement primitive.

#### Graph representation of traversable space

Given the transition function $\mathcal {T}$, the level map $\mathcal {M}$ is encoded as a weighted directed graph *G = (V, E, ω )*, where the node set *V* comprises all passable tiles:

$$V = \bigl \{\, v_{i,j} \;\mid \; \phi (M_{i,j}) = 0,\; 1 \le i \le H,\; 1 \le j \le W \,\bigr \},$$

and a directed edge $(v_{i,j},\, v_{k,l}) \in E$ exists if and only if the corresponding transition is physically feasible:

$$E = \bigl \{\,(v_{i,j},\, v_{k,l}) \;\mid \; \mathcal {T}\bigl ((i,j),\,(k-i,\,l-j)\bigr ).{\text {feasible}} = 1\,\bigr \},$$

with each edge weighted by the associated traversal cost $\omega (v_{i,j}, v_{k,l})$.

Since every edge in *G* is guaranteed to follow a collision-free trajectory under the full linear interpolation check, the graph encodes only physically realizable player movements, enabling efficient reachability queries without requiring full game simulation.

#### Reachability analysis via Dijkstra’s algorithm

Given the graph *G = (V, E, ω )*, playability is determined by computing the minimum-cost path from the set of valid start nodes $V_{\text {start}}$ to the set of valid goal nodes $V_{\text {goal}}$, defined respectively as:

$$V_{\text {start}} = \bigl \{\, v_{i,\,1} \;\mid \; \text {grounded}(i, 1) \,\bigr \}, \qquad V_{\text {goal}} = \bigl \{\, v_{i,\,W} \;\mid \; \phi (M_{i,W}) = 0 \,\bigr \}.$$

Dijkstra’s algorithm^18^ is applied to compute the shortest path from each candidate $v_s \in V_{\text {start}}$ to each candidate $v_g \in V_{\text {goal}}$. The level is classified as playable if and only if at least one feasible path exists:

$$\text {Playable}(\mathcal {M}) \;\triangleq \; \exists \; v_s \in V_{\text {start}},\; v_g \in V_{\text {goal}} \;:\; d_G(v_s,\, v_g) < \infty ,$$

where $d_G(v_s, v_g)$ denotes the shortest-path distance in *G* under the edge weights *ω*. If no such path exists across all pairs $(v_s, v_g) \in V_{\text {start}} \times V_{\text {goal}}$, the level is labeled non-playable.

**Fig. 6:**
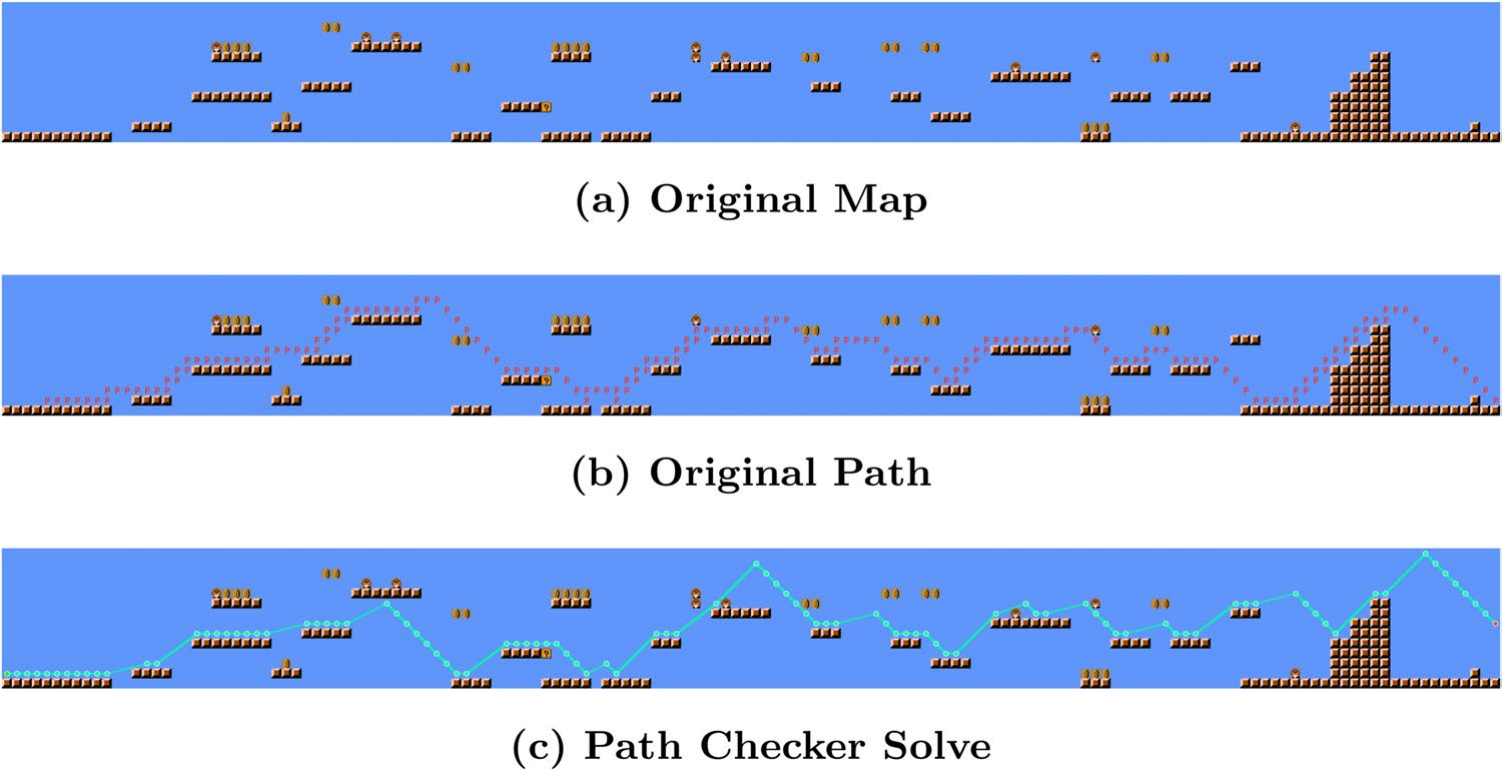
Example of a verified traversal path computed by the physics-constrained pathfinding procedure.

This formulation provides a rigorous, physics-aware criterion for playability that is both computationally efficient and grounded in the actual movement capabilities of the player character. By enforcing collision-free trajectories through full linear interpolation and global traversal feasibility through shortest-path analysis, the verification stage ensures that all retained levels satisfy a necessary condition for meaningful gameplay. An example of a verified traversal path computed by this procedure is illustrated in Fig. 6.

## Results

This section presents the experimental results of the proposed approach across four main components. First, the clustering analysis of the human gameplay trajectories is reported, including the resulting cluster centroids and their assigned skill-level labels. Second, the classification performance of the trained model is evaluated on a held-out test set, with per-class metrics and a confusion matrix reported. Third, the playability verification outcomes are presented across both the original and LLM-modified levels, followed by qualitative examples illustrating the behavior of the modification pipeline. Fourth, a structural design metric analysis is presented, providing objective evidence that the modification pipeline produces level content that is statistically aligned with the targeted skill labels.

### Human gameplay trajectory clustering

Table 3 reports the mean values of the most discriminative features across the three clusters identified by K-means, along with their assigned skill-level labels.

**Table 3:** K-means cluster centroids and assigned skill-level labels based on key behavioral features.

Label	$\overline{v}_x$	$\overline{\text {pos}\_\text {x}}$	$\overline{\text {sw}\_\text {rate}}$
Beginner	7.91	30.67	0.56
Normal	18.01	434.40	0.17
Expert	30.70	578.87	0.17

The clustering yielded a silhouette score of 0.189, which is indicative of the inherently continuous nature of skill-level transitions in human gameplay, where boundaries between categories are gradual rather than sharply defined.

### Human behavior classification

The XGBoost classifier was trained to categorize players into three skill levels: Expert, Normal, and Beginner. The model was evaluated on a held-out test set of 5,097 samples, with 1,699 samples per class following SMOTE-based balancing. The classifier achieved an overall accuracy of 97.82%, demonstrating strong discriminative capability across all three player categories.

Table 4 reports the per-class performance metrics. All three classes achieved an F1-score of 0.98, with precision and recall consistently at or above 0.97 across all categories. The macro-averaged precision, recall, and F1-score were all 0.98, confirming that the model performs uniformly well regardless of player skill level.

**Table 4:** Per-class classification performance of the XGBoost player behavior model.

Class	Precision	Recall	F1-Score	Support
Expert	0.97	0.98	0.98	1,699
Normal	0.98	0.97	0.98	1,699
Beginner	0.98	0.98	0.98	1,699
Macro Avg	0.98	0.98	0.98	5,097
Weighted Avg	0.98	0.98	0.98	5,097

Table 5 presents the confusion matrix of the model. The dominant diagonal values confirm strong per-class accuracy, while the low off-diagonal counts indicate that misclassifications are rare and largely confined to neighboring skill levels, suggesting that the feature representation captures meaningful behavioral distinctions between player categories.

**Table 5:** Confusion matrix for the XGBoost player behavior classification model.

	**Predicted Label**			
**Actual Label**	Expert	Normal	Beginner	Total
Expert	**1,670**	6	23	1,699
Normal	35	**1,650**	14	1,699
Beginner	11	22	**1,666**	1,699

### Level playability verification

The playability verification procedure was applied in two stages. In the first stage, the checker was run on the 15 original Mario levels to validate the reliability of the verification pipeline. Of these, 12 levels were confirmed as playable and 3 were found to be non-playable, yielding a baseline playability rate of 80.0%.

In the second stage, six skill-conditioned prompts were applied to the original levels, with two prompts per skill level (Beginner, Normal, Expert), each targeting a distinct structural modification objective. Every prompt was applied to a randomly selected chunk of each level, producing a total of 90 modification runs across 15 levels. Table 6 lists the prompts used in this experiment.

**Table 6:** Skill-conditioned prompts used for LLM-based level modification.

Skill	ID	Prompt
Beginner	B1	Ensure a safe path: remove all enemies and gaps, creating a continuous flat ground surface to prevent any falling risk.
	B2	Add a reward trail of coins at row 11 across the entire chunk and place question blocks at reachable heights to guide the player forward.
Normal	N1	Add 2 isolated enemies in open spaces and place a pipe structure that the player must jump over.
	N2	Replace one ground section with a small 2-tile gap and place a question block with a coin above the landing spot to reward the jump.
Expert	E1	Create a hazard bottleneck by placing 3–4 enemies in a tight cluster and removing 3 ground blocks immediately after them to force a high-precision jump.
	E2	Introduce a precision platforming section: replace a segment of ground with three 1-tile pillars and place a cannon on the center pillar.

Of the 90 modification runs, the pipeline successfully generated modified outputs for 85 chunks, while the remaining 5 runs produced no change and were excluded from further analysis. Playability was subsequently evaluated at two granularities: at the complete level granularity, where the modified chunk is reassembled into the full level map and subjected to end-to-end path verification, and at the isolated chunk granularity, where only the modified segment is evaluated independently. The results are summarized in Table 7.

**Table 7:** Playability verification results across original and modified levels.

Condition	Total	Playable	Not playable	Playability rate
Original levels	15	12	3	80.0%
Modified levels (full)	85^a^	63	22	74.12%
Modified chunks (isolated)	85^a^	71	14	83.53%

Out of 90 total modification attempts, 5 runs produced no change and were excluded from playability analysis

The chunk-level playability rate of 83.53% exceeds the complete level rate of 74.12%, which is an expected outcome: a modified chunk may be internally traversable while introducing a structural discontinuity at the boundary with an adjacent unmodified chunk, causing the reassembled level to fail the end-to-end path verification. Both rates remain competitive with the 80.0% baseline of the original unmodified levels, demonstrating that the skill-conditioned modification pipeline produces structurally valid chunks without significantly compromising overall level navigability.

### Structural design metric analysis

To provide objective evidence that the two-stage LLM pipeline successfully restructures level content in alignment with the targeted skill labels, three established structural design metrics were computed on all 71 playable modified chunks. Leniency and Action Density are operationalized from the metric framework proposed by Canossa and Smith^20^, while Topographical Roughness follows the formulation adopted in search-based procedural content generation^21^. Metrics were computed exclusively on chunks that passed the physics-constrained playability verification, ensuring that structurally malformed outputs do not contaminate the analysis.

**Leniency** quantifies the balance between rewarding and hazardous elements relative to the chunk width *W*, following the conceptual framework of Canossa and Smith^20^. Let *R* denote the count of reward tiles (question blocks ?, Q and coins o), *H* the count of hazard tiles (enemies E and cannons B, b), and *G* the number of gap columns, defined as columns containing no solid tile. Leniency is then:

18 $$\begin{aligned} \text {Leniency} = \frac{R - H - G}{W} \end{aligned}$$

Higher values indicate a more forgiving chunk; lower values indicate a more hazardous one.

**Topographical Roughness** measures terrain variation by computing the mean absolute height difference between adjacent columns^21^. Let $h_j$ denote the height of the highest solid tile in column *j*, measured in rows from the bottom. Roughness is defined as:

19 $$\begin{aligned} \text {Roughness} = \frac{1}{W-1} \sum _{j=2}^{W} \left| h_j - h_{j-1} \right| \end{aligned}$$

Higher values correspond to more irregular terrain requiring more precise navigation.

**Action Density** quantifies the concentration of interactive challenge elements per unit width, extending the density metric of Canossa and Smith^20^ to focus specifically on action-requiring tiles. Let *A* denote the count of action-requiring tiles (enemies E, pipes $\texttt {<>}$, cannons B, b, and question blocks ?, Q), and *G* the number of gap columns. Action Density is defined as:

20 $$\begin{aligned} \text {Action Density} = \frac{A + G}{W} \end{aligned}$$

Higher values indicate a denser concentration of obstacles and interactive elements.

#### Descriptive statistics

Table 8 reports the mean and standard deviation of each metric across the three skill groups. Leniency decreases monotonically from Beginner (*μ = 0.3176*) to Normal (*μ = -0.1441*) to Expert (*μ = -0.1753*), indicating that Beginner chunks contain proportionally more rewards and fewer hazards relative to Expert and Normal chunks. Topographical Roughness increases monotonically from Beginner (*μ = 0.6652*) to Normal (*μ = 1.0785*) to Expert (*μ = 1.4526*), reflecting progressively greater terrain variation at higher skill levels. Action Density follows the same ordering, with Beginner chunks recording the lowest density (*μ = 0.2244*) and Normal chunks the highest (*μ = 0.5792*), confirming that interactive challenge elements are more densely packed in chunks targeting more skilled players.

**Table 8:** Per-skill descriptive statistics of structural design metrics computed on 71 playable modified chunks.

Metric	Skill	N	Mean	Std	Min	Median	Max
Leniency	Beginner	22	0.3176	0.4136	*-0.1212*	0.0829	1.0000
	Normal	24	*-0.1441*	0.1522	*-0.3793*	*-0.1571*	0.2308
	Expert	25	*-0.1753*	0.1793	*-0.4857*	*-0.1667*	0.2692
Topographical Roughness	Beginner	22	0.6652	0.6014	0.0000	0.6720	1.9412
	Normal	24	1.0785	0.6081	0.0000	0.9821	2.4167
	Expert	25	1.4526	0.5924	0.4000	1.5333	2.7419
Action Density	Beginner	22	0.2244	0.2443	0.0000	0.1100	0.7576
	Normal	24	0.5792	0.3622	0.2308	0.4627	1.5312
	Expert	25	0.4890	0.3671	0.1200	0.4324	1.6857

#### Statistical testing

To determine whether the observed differences across skill groups are statistically significant, the non-parametric Kruskal-Wallis test was applied to each metric, as the data cannot be assumed to follow a normal distribution. The results, reported in Table 9, confirm statistically significant differences across all three metrics (*p < 0.001*). Pairwise post-hoc comparisons were subsequently conducted using the Mann-Whitney U test, with results reported in Table 10.

**Table 9:** Kruskal-Wallis test results across all three skill groups.

Metric	H-statistic	*p*-value	Result
Leniency	29.9525	0.000000	Significant
Topographical roughness	16.5208	0.000259	Significant
Action density	15.6172	0.000406	Significant

**Table 10:** Pairwise Mann–Whitney U post-hoc test results.

Metric	Comparison	*p*-value	Result
Leniency	Beginner vs normal	0.000003	Significant
	Beginner vs expert	0.000001	Significant
	Normal vs expert	0.502705	Not significant
Topographical roughness	Beginner vs normal	0.023395	Significant
	Beginner vs expert	0.000134	Significant
	Normal vs expert	0.026319	Significant
Action density	Beginner vs normal	0.000487	Significant
	Beginner vs expert	0.001533	Significant
	Normal vs expert	0.218674	Not significant

The post-hoc results reveal that Beginner chunks are significantly different from both Normal and Expert chunks across all three metrics, confirming that the pipeline reliably produces structurally distinct content for the lowest skill level. For Topographical Roughness, all three pairwise comparisons reach significance, indicating a clean monotonic separation across the full skill spectrum. For Leniency and Action Density, the Normal versus Expert comparison does not reach significance (*p = 0.502705* and *p = 0.218674* respectively). The implications of these findings are discussed in “Discussion” section.

### Qualitative level modification examples

**Fig. 7:**
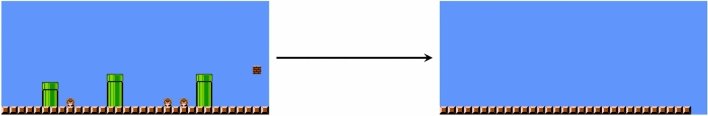
Modification example 1 (Beginner, B1): Ensure a safe path: remove all enemies and gaps, creating a continuous flat ground surface to prevent any falling risk.

**Fig. 8:**
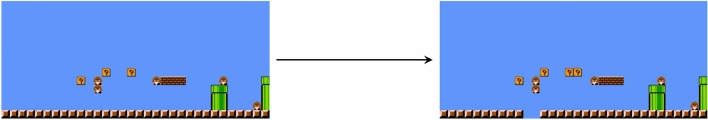
Modification example 2 (Normal, N2): Replace one ground section with a small 2-tile gap and place a question block with a coin above the landing spot to reward the jump.

**Fig. 9:**
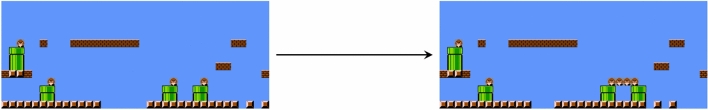
Modification example 3 (Expert, E1): Create a hazard bottleneck by placing 3–4 enemies in a tight cluster and removing 3 ground blocks immediately after them to force a high-precision jump.

**Fig. 10:**
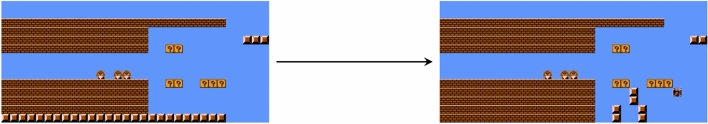
Modification example 4 (Expert, E2): Introduce a precision platforming section: replace a segment of ground with three 1-tile pillars and place a cannon on the center pillar.

**Fig. 11:**
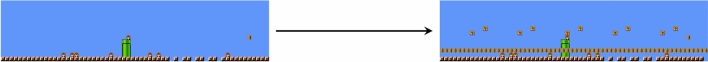
Modification example 5 (Beginner, B2): Add a reward trail of coins at row 11 across the entire chunk and place question blocks at reachable heights to guide the player forward.

To further illustrate the behavior of the LLM-based modification pipeline, Figs. 7, 8, 9, 10, and 11 present five examples of level chunks before and after modification, along with the prompt used in each case. Each example shows the original chunk on the left, the applied prompt in the center, and the resulting modified chunk on the right.

## Discussion

### Cluster quality and label validity

K-means clustering was applied to a human gameplay dataset of 8,072 samples, yielding a silhouette score of 0.189. This is an expected outcome given that gameplay behavior across skill levels is not categorically distinct – players share the same action space, level structure, and physical state transitions, causing the high-dimensional feature space to appear dense and overlapping. Nevertheless, centroid statistics reveal clear monotonically ordered differences in the features that matter: $\overline{v}_x$ increases consistently from beginner to expert, and $\overline{\text {sw\_rate}}$ drops sharply, indicating more decisive input behavior at higher skill levels. The cluster-to-label mapping was therefore grounded in $\overline{v}_x$ as the single most discriminative behavioral signal in the dataset. That said, two limitations apply here: the human trajectories were collected through deliberate skill mimicry rather than organic gameplay, and the label mapping relies on a single feature that may not generalize to contexts where velocity is not the primary discriminating factor.

### Interpretation of classification results

The XGBoost classifier achieved an overall accuracy of 97.82% with a macro-averaged F1-score of 0.98. Prior to incorporating human gameplay data, the classifier exhibited a strong bias toward the expert class due to the agent-generated dataset being predominantly high-performing. The addition of human trajectories corrected this imbalance by introducing behavioral diversity, improving distributional coverage rather than merely increasing sample size. The confusion matrix further supports this: misclassifications are rare and confined to adjacent skill levels, confirming that the classifier has internalized a coherent ordinal structure consistent with the underlying behavioral reality. Nevertheless, the quality of the learned boundaries remains contingent on the representativeness of the human-labeled data, which, as noted, was collected under controlled mimicry conditions.

### Playability preservation under LLM-based modification

The modified levels achieved a full-level playability rate of 74.12% and an isolated-chunk playability rate of 83.53%, compared to a baseline of 80.0% for the original unmodified levels, as detailed in Table 7. The full-level rate falls below the baseline primarily due to chunk boundary discontinuities, where a modified chunk that is internally traversable may nevertheless introduce a structural break at its junction with an adjacent unmodified chunk. The chunk-level rate of 83.53% exceeds the baseline, confirming that individual modified chunks are on average more structurally sound than those in the original unmodified levels, and that the observed full-level degradation is an integration artifact rather than a generation failure. Of the 90 modification attempts, 5 were removed due to unchanged outputs or API failures. Two additional limitations should be noted: the playability checker is a static rule-based tool rather than a simulation-based verifier, making it susceptible to false negatives that likely cause both rates to be slightly underestimated. Furthermore, the framework was evaluated exclusively on Super Mario Bros. levels, and its generalizability to other platformer games or level structures remains to be validated.

### Structural alignment with skill labels

The metric analysis provides objective evidence that the pipeline systematically restructures level content in alignment with the targeted skill label rather than modifying levels arbitrarily. Across all three metrics, Beginner chunks are significantly separated from both Normal and Expert chunks, confirming that the pipeline reliably produces safer, flatter, and less action-dense content for lower-skill players. Topographical Roughness exhibits a clean monotonic ordering across all three skill levels with all pairwise comparisons reaching significance, while Leniency records the strongest overall separation (*H = 29.95*, *p < 0.001*). These results are consistent with the established role of these metrics as proxies for level difficulty and navigational complexity^20,21^.

The one limitation concerns the Normal versus Expert distinction. For Leniency and Action Density, the pairwise comparison between these two groups does not reach significance (*p = 0.502705* and *p = 0.218674* respectively). This is attributable to the Expert prompts primarily targeting hazard clustering and terrain irregularity rather than increasing overall element count, causing aggregate tile-level statistics for both groups to remain similar. Topographical Roughness, being sensitive to spatial terrain distribution rather than element counts, successfully separates Normal from Expert (*p = 0.026319*), supporting this interpretation. Strengthening prompt-level differentiation between Normal and Expert represents a clear direction for future refinement.

## Conclusion

This work presented an adaptive Mario level modification approach that combines player skill classification with prompt-based generative content transformation. A hybrid behavioral dataset was constructed by merging agent-generated trajectories from PPO-trained agents at three skill levels with human gameplay data labeled through clustering. A classifier trained on this dataset achieved an accuracy of 97.82%, confirming that the behavioral state representation captures sufficient discriminative signal to reliably categorize players. The classification output drives a two-stage LLM pipeline that expands a skill-conditioned prompt into a structured modification instruction applied to the current level chunk, with a physics-constrained Dijkstra-based verifier ensuring traversability of all modified levels. Evaluated across 15 Super Mario Bros. levels, the pipeline achieved a full-level playability rate of 74.12% and an isolated-chunk playability rate of 83.53%, both competitive with the 80.0% baseline of the original unmodified levels, confirming that structural integrity is preserved under targeted modification. A structural design metric analysis further demonstrated statistically significant differences in Leniency, Topographical Roughness, and Action Density across the three skill groups, providing objective evidence that the pipeline reliably produces content aligned with the intended skill label. Future work includes refining prompt instructions to reduce generation failures and improve Normal versus Expert differentiation, introducing a repair mechanism for self-correction of invalid chunks, conducting controlled user studies to evaluate the impact of adaptive modifications on player engagement and perceived difficulty, extending the approach to other platformer games to validate its generalizability beyond Super Mario Bros., and applying the pipeline to puzzle-driven games such as The Legend of Zelda, where LLM-based chunk modification could directly leverage symbolic reasoning to preserve key and lock dependencies and puzzle logic across room graphs.

## Data Availability

The Mario level data, the human gameplay dataset collected in this study, and the source code are publicly available at the following Zenodo repository: https://zenodo.org/records/20844868

## Code availability

The source code for the full adaptive level modification pipeline, including the two-stage LLM modification framework, the XGBoost-based skill classifier, and the physics-constrained playability verifier, has been deposited on Zenodo at https://doi.org/10.5281/zenodo.20844868 for reproducibility.
